# A Systematic Review of the Stability of Extemporaneous Pediatric Oral Formulations

**DOI:** 10.1155/2021/8523091

**Published:** 2021-12-15

**Authors:** Anteneh Belayneh, Zenaw Tessema

**Affiliations:** Department of Pharmacy, College of Health Sciences, Debre Markos University, Debre Markos, Ethiopia

## Abstract

**Background:**

Extemporaneous compounding is a pharmacy practice to produce suitable pharmaceutical preparations when there are no commercially available, licensed, and age-specific dosage forms. Compared to the use of authorized drugs, these preparations have significant risks. Stability issues are one of the major concerns during the preparation of extemporaneous formulations.

**Aim:**

The aim of this work was to study the stability of pediatric extemporaneous formulations of commercially available conventional solid dosage forms by reviewing systematically the currently available stability studies.

**Method:**

Articles were searched in the databases of the Web of Science, PubMed, Scopus, EMBASE, Cochrane Library, and Google Scholar. From all the searched articles, a total of 28 experimental studies reporting the stability of oral pediatric extemporaneous formulations were included based on the inclusion criteria. Oral extemporaneous formulations from commercially available dosage forms and pure drugs were considered. According to the United States and British Pharmacopeia (USP and BP), most extemporaneous formulations are accepted as chemically stable if they maintain ≥90% of the original drug amount, physically stable if there is no apparent change in physical property, and microbiologically stable if there is no growth of microorganisms in prepared formulations. *Finding*. In this study, most extemporaneous pediatric oral formulations were chemically, physically, and microbiologically stable and retained more than 90% of the initial content. Very few studies did not include either a physical stability test or a microbiological stability test.

**Conclusion:**

According to this systematic review, the chemical and physical instabilities as well as microbial growth on pediatric oral extemporaneous formulations are very rare in published experimental studies. Most studies show that extemporaneous preparations are stable at the ICH recommended storage conditions and duration. Generally, extemporaneously prepared oral formulations will be the promising option for child medications.

## 1. Introduction

### 1.1. Background

The shortage of suitable pharmaceutical dosage forms specifically designed for children is the major challenge for pediatric treatment. Most FDA-approved adult pharmaceutical dosage forms are not appropriate formulations for pediatric use. The lack of sufficient information on pediatric administration often leads to the unauthorized use of adult preparations by healthcare professionals [1]. In some cases, there are no licensed or substitute products that fully meet the clinical needs of specific patients, so it is necessary to temporarily prepare a limited number of customized products for individual patients. About 15% to 80% of all drugs used by hospitalized children are either unlicensed or used outside of the product's license specifications (“off-licence”) [2].

Extemporaneous formulation describes the use of traditional compounding techniques by pharmacists to manipulate various drugs and chemical ingredients to produce suitable drugs when commercial forms are not available. These techniques are widely used in the practice of pediatric pharmacy. Most approved oral medications for adults are provided in tablet or capsule form, usually in a single adult dose form or in a liquid form that is not suitable for infants. However, the dose size of pediatric medications should change proportionally to body surface area and body weight during childhood. Also, most of the pediatric population cannot swallow pills, capsules, and other conventional dosage forms. To prevent the inappropriate use of unlicensed and unapproved adult medications, pharmacists will prepare suitable pediatric preparations [3]. This can be accomplished by grinding approved adult solid dosage forms such as tablets or by using capsule contents (powder and granules). Then, the powder can be prepared in the form of oral solution or suspension preparations using appropriate excipients and a suitable vehicle to produce, or it can be diluted into lower strength solid dosage forms using inert diluents. Sometimes the tablets are segmented into lower portions (half or a quarter) to get a suitable dosage unit for children [2].

### 1.2. Stability of Extemporaneous Pediatric Formulation

The physical, chemical, and microbiological stability should be considered during the quality assessment of extemporaneous preparation. It is very important to meet the storage conditions indicated on the label. Even if it has been proven that a given pharmaceutical preparation has sufficient physical, chemical, and microbiological stability, the bioavailability and palatability of the formulation may not be proven. Few pharmaceutical formulations are supported by evidenced data that determine sufficient absorption curves and/or bioequivalence with licensed formulations. Insufficient access to raw materials and equipment is also another concern during the compounding of good quality extemporaneous pharmaceutical products. In order to reduce degradation and deterioration, the maximum shelf life of the product is 28 days, unless the product is chemically unstable, so the shelf life is based on the stability of the respective products. Stability studies of these formulations are usually conducted in a short period of time. The lack of stability data limits the availability of many pediatric drugs. The candidate formulations available for extemporaneous preparations are highly dependent on the accessibility of stability data and the ingredients required for the compounding [2].

The objective of this study was to systematically review the stability of pediatric extemporaneous pharmaceutical formulations. The specific aim of this study was first to assess the stability of oral pediatric extemporaneous formulations by reviewing the currently available experimental literature and to provide evidence-based or best practice guidance about the chemical, physical, and microbiological stability of extemporaneous oral preparations of medicines for pediatrics. This helps policy makers and clinical practitioners who use extemporaneous preparations for pediatrics. Pharmacists, the main concerned professionals of pharmaceutical compounding, will benefit from the findings of this systematic review.

## 2. Methods

### 2.1. Literature Searching Strategy

The systematic reviews follow the Cochrane Collaboration guidelines, and we record the results according to the PRISMA guidelines for systematic reviews and meta-analysis preferred reporting project (PRISMA flowchart) [4]. We searched related experimental works of literature according to the study objectives from reliable databases of the Web of Science, PubMed, Scopus, EMBASE, Cochrane Library, and Google Scholar databases, written in English from June 1, 2021, to July 5, 2021. We combined the search strategy for free text terms and exploited the MESH title for the topics “Extemporaneous formulation OR Extemporaneous preparation OR Extemporaneous compounding,” “stability,” and “Peadiatric OR child OR Neonate OR Infant” using the Boolean operators like “AND” or “OR.”

### 2.2. Study Selection and Eligibility Criteria

All currently online available experimental works conducted on the stability of extemporaneous pediatric formulations were included in the study. The articles and records which had no stability data, not focused on pediatric formulations, not focused on oral formulations, and articles without an informative abstract or full document were excluded from the study.

#### 2.2.1. Eligibility Criteria

*(1) Inclusion Criteria*. Two researchers (AB and ZT) independently and carefully reviewed the content of each retrieved article. Finally, documents that meet the following criteria are included in the study.

  Population: studies on the stability of pediatric extemporaneous formulations were included
  Study area: all articles were included irrespective of the specific study area and year of the study
  Study design: original experimental works of literature which have data on the stability of pediatric extemporaneous preparations were eligible
  Language: documents published in English were considered
  Publication condition: documents that fulfill the inclusion criteria were considered regardless of their publication status

*(2) Exclusion Criteria*. Two independent reviewers performed blind data extraction after evaluating the abstract and full text of the literature. After reading the full text and abstract, articles with methodological issues were excluded by two independent researchers. Due to incomplete data, inaccessible full-text articles were not included in the review.

*(3) Data Extraction*. Using the previously tested data extraction format, the researchers extracted the necessary data. Data extracted from included studies are as follows: author, study area, method of chemical stability test, source drug for extemporaneous formulation, storage condition of extemporaneous formulation, chemical stability result, physical stability result, and microbiological stability study. Any differences between the two authors on data extraction are resolved through discussion.

## 3. Results and Discussion

We reviewed all available studies which focused on the chemical, physical, and microbiological stability of pediatric oral extemporaneous formulations. We considered whether or not instability problems occurred in such preparations. A limitation of our review is that the protocol was not previously registered. We found encouraging research on the stability of many drugs for pediatric oral formulations.

### 3.1. Selection of Included Studies

**Figure 1:**
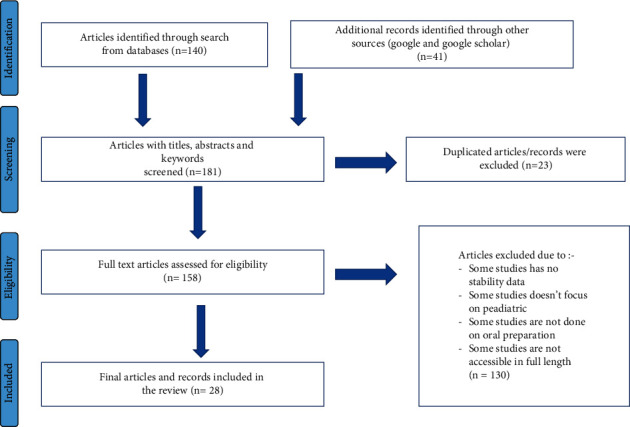
PRISMA diagram showing studies utilized for systematic review of stability of pediatric oral extemporaneous oral formulations.

The search for the database has brought a total of 181 research items. Duplicate research (*n* = 23) was eliminated through its titles and summaries. The research approved by the abstract review was also examined with its title. Finally, a total of 28 studies (experimental articles) were included in this systematic review (Figure 1).

### 3.2. Source of Pediatric Extemporaneous Oral Liquid Formulations

In this systematic review, commercially available tablets and capsules are the most commonly used sources for pediatric extemporaneous preparations. Pure drugs, injectable preparations, and pellets are also used as sources of pediatric extemporaneous preparations. The results are consistent with other studies (Table 1). Research conducted at the Malta Hospital shows that most improvised pediatric compounding is made by converting capsules and tablets into oral liquids or powder. Others are made from active ingredients in bulk, such as oseltamivir powder to make an oseltamivir phosphate solution [2]. Preparation of children's oral medicines is subject to much variation in hospitals throughout Europe, and there is little harmonization of formulations or information on the stability of products. The European Union could be the focus for improving the availability of appropriate authorized medicines for children and ensuring that when extemporaneous preparation is necessary, it is of a common high standard [3].

### 3.3. Methods for Stability Testing

For this study, high-performance liquid chromatography (HPLC) is the frequently used method to check the chemical stability of most extemporaneous preparations. The UV-spectrophotometer and other methods are also used rarely to check the stability of these preparations (Table 1). HPLC and UV-spectrophotometer are the recommended methods for stability test of pharmaceutical formulations by all pharmacopeias including United States Pharmacopeia (USP) and British Pharmacopeia (BP) [7]. The stability of the peaks of the analyte and the degradation product that are completely separated from each other is indicative of the chromatogram of the HPLC method. [8].

### 3.4. Storage Condition of Pediatric Extemporaneous Formulations

During storage, pharmaceuticals are prone to physical and chemical degradation. These degradations may change the pharmacological properties of the drug, reducing its benefits and increasing its harmful effects. The physical factors which affect the stability of the drug are light, solvent, heat, oxygen, and humidity [9].

In this review, most of the pediatric extemporaneous formulations were stored at temperatures of 4, 25, and 40°C with and without light before testing. Some were stored at room temperature until they were tested. The storage duration for the test was varied from 24 hours to 150 days (Table 1). All storage conditions comply with the ICH guideline which focuses on the storage conditions of pharmaceuticals for the purpose of stability testing of APIs [10].

### 3.5. Chemical Stability of Pediatric Oral Extemporaneous Liquid Formulations

Stability studies to ensure pharmaceutical product safety, quality, and efficacy are preserved throughout the shelf life and are considered as a precondition for approval of any pharmaceutical preparations. Experimental stability studies should be done in a well-organized manner according to the World Health Organization (WHO) and International Conference on Harmonization (ICH) guidelines. Stability refers to the degree to which a product maintains the same characteristics during its storage and use within the specified limits. Each drug maintains chemical integrity and labeled efficacy within the specified range [8].

More than 96% of pediatric oral extemporaneous liquid formulations in this review are stable at all storage conditions (4°C, 25°C, 40°C, and room temperature). They retain more than 90% of their initial content of the active drug after the storage duration. But, some drugs are unstable in some specific conditions. For instance, nifedipine and pyridoxal phosphate oral liquid extemporaneous preparations were degraded in the exposure of light. Amlodipine was also degraded at 25 and 40°C. From twenty-eight pediatric oral extemporaneous formulations, only three formulations showed chemical degradation (Table 1).

The USP, BP, and European Pharmacopeia have established that the acceptable range of most compounded preparations is typically ±10%, or within the range of 90.0%–110.0%. Even for some drugs, if they retain 85% of their original content, it is acceptable [8].

### 3.6. Physical Stability of Pediatric Oral Extemporaneous Liquid Formulations

The appearance, consistency, uniformity of content, solution clarity, moisture content, particle size and shape, pH value, and integrity of pharmaceutical packaging may change, which may affect its stability. Such physical changes can be caused by shock, vibration, wear, and temperature fluctuations (such as freezing, thawing, or shearing) [37]. To conclude a pharmaceutical product as physically stable, the original physical properties, including appearance, palatability, uniformity, dissolution, and suspendability, must be retained [8].

In this review, almost all (98.9%) of the extemporaneous pediatric formulations are physically stable at all storage conditions. There were no apparent changes in color, odour, viscosity, redispersibility, and pH. But some of the literature (7 from 28) have no data for physical stability (Table 1).

### 3.7. Microbiological Stability of Pediatric Oral Extemporaneous Liquid Formulations

The stability of a pharmaceutical product can also be affected because of microbiological changes such as the growth of microorganisms in nonsterile products and changes in preservative efficacy. Microbiological tests include sterility, preservative efficacy, and microbial count as applicable. Resistance to microbial growth is retained according to the specified requirements [37].

We also discuss the factors that affect microbial contamination in popular dosage forms (e.g., tablets, sterile products, cosmetics). When these products are contaminated, the microorganisms can cause changes. The effects range from mild changes (e.g., discoloration, texture alteration) to severe effects (e.g., changes in activities, toxicity). In this study, most pediatric oral extemporaneous preparations in this review are stable (no growth of microorganisms at the storage temperature during the storage conditions). But some studies do not include microbiological stability study (Table 1)).

## 4. Conclusion

There are severe shortage of commercial drugs suitable for children, due to this there is a need for extemporaneous oral preparations. Stability study data are very crucial for hospital pharmacists to confirm the safety and quality of the dispensed extemporaneous preparations, especially for pediatric patients. To be used as an alternative for commercial products and to be therapeutically safe and effective, the extemporaneous pharmaceutical preparation must be physically, chemically, and microbiologically stable.

According to this systematic review, several experimental studies showed that the chemical and physical instabilities and microbial growth on pediatric oral extemporaneous formulations are very rare. Most studies revealed extemporaneous preparations are stable at the ICH recommended storage conditions and duration. It is recommended that an expiry date of a maximum of one month (or less if advised in the published study or if antimicrobial preservatives cannot be used) is applied to all extemporaneous formulations. This will encourage to use regular fresh preparation and help to assure effectiveness and safety. It also allows the practitioner to regularly review the patient's use of the preparation.

Generally, extemporaneously prepared oral formulations (medicines) from commercially available tablets, capsules, powders, and other dosage forms will be the promising option for pediatrics.

## Figures and Tables

**Table 1:** The stability of oral liquid extemporaneous preparations compounded from commercially available formulations.

Author, year (references)	Study area	Method of chemical stability test	Source drug for extemporaneous formulation	Storage conditions of extemporaneous formulation	Stability result of oral extemporaneous liquid formulation		
					Chemical stability result	Physical stability result	Microbiological stability study
Alemón-Medina et al., 2015 [5]	Mexico	UPLC-UV	Metformin tablet	25 ± 2°C by exposing to light and dark, 4°C in the dark, and 40°C in the dark for 30 days	Stable at all storage conditions (>90% of the initial amount) (i) No change in pH	Stable (no change in color, no foreign particles, and no turbidity)	—
Alemon-Medina et al., 2012 [6]	Mexico	UPLC-UV	Glucophage® tablets (metformin)	25 ± 2°C by exposing to light and dark, 4°C in the dark, and 40°C in the dark for 30 days	Stable (>90% of its initial content)	Physically stable for 30 days	Stable (no apparent bacterial or yeast growth)
Ali et al., 2016 [11]	The United Arab Emirates	UV-spectrophotometer	Furosemide tablet	4 and 25°C for 90 days	Stable (>98%)	Stable (no change in viscosity, color, odour, and dispersibility)	Stable (no apparent bacterial)
Boscolo et al., 2020 [12]	Argentina	HPLC	Omeprazole pellets	Stored in 4°C and 25°C temperature for 150 days	(i) At 4°C, stable for at least 150 days (ii) Stable for 14 days at 25°C (iii) No change in pH	(i) Physically stable (no change in color, redispersibility, and flavor)	Stable (no contamination of E. coli, fungi, and yeast was observed)
Buontempo et al., 2010 [13]	Argentina	HPLC	Carvedilol tablet	4, 25, and 40°C for 56 days	(i) Stable at all temperatures (>99%)	—	—
Buontempo et al., 2013 [14]	Argentina	HPLC	Clobazam tablets	4 and 25°C for 56 days	Stable (>98%)	Stable	—
Casas et al., 2015 [15]	Spain	HPLC	Pure drug of enalapril and captopril	5, 25, and 40°C for 90 days	(i) Stable only for 50 days at 5^0^C	(i) Stable in consistency	—
Freed et al., 2005 [16]	USA	HPLC	Accupril® tablets (quinapril)	−5°C for 28 days	Stable (≥90%)	—	—
García et al., 2017 [17]	Argentina	UV-spectrophotometry	Benznidazole tablets	5 and 25°C for 90 days	Stable (>99%) and no pH change	(i) No detectable changes in color, odour, and viscosity	Stable
Han et al., 2006 [18]	UK	—	Tacrolimus pure drug	22–26°C for 56 days	—	Stable	Stable
Helin-Tanninen, 2010 [19]	Finland	HPLC	Nifedipine tablet	At 22°C and 6°C for 28 days	(i) Stable when protected from light, whereas on exposure to light, nifedipine degrades rapidly	—	—
Juárez Olguín et al., 2008 [20]	Mexico	HPLC	Propafenone tablet	15 ± 5°C and at refrigeration (3–5°C) for 90 days	Stable (>90%)	No color and odour change	Stable
Klovrzová, 2016 [21]	Czech Republic	HPLC	Pure sotalol	Room temperature and in a refrigerator for 180 days	Stable (>95%)	—	Stable
Klovrzová et al., 2013 [22]	Czech Republic	HPLC	Propranolol tablet	5 ± 3°C and 25 ± 3°C for 180 days	Stable at both storage temperatures	—	Stable
Kučerová et al., 2019 [23]	Czech Republic	Ultra-HPLC method with UV detection	Pure omeprazole drug	4°C for 15 days	Stable	—	—
Lafuente et al., 2019 [24]	Argentina	HPLC	Indomethacin injection	Room temperature for 17 days	Stable	Stable (no detectable changes in color, odour, and/or flavor)	Stable
Lin et al., 2021 [25]	Taiwan	HPLC	Propafenone hydrochloride tablets	2–8°C and room temperature for 90 days	Stable	Stable	Stable
Mahmoud et al., 2014 [26]	Saudi	UV-spectrophotometer	Spironolactone tablets	Room temperature for 35 days	Stable (>90%)	Stable (no appearance change)	Stable
Mendes et al., 2013 [27]	Brazil	HPLC	Furosemide, hydrochlorothiazide, and spironolactone tablets	25 ± 2°C and 5 ± 3°C for 7 days	Stable, except hydrochlorothiazide	Stable	Stable
Mohamed‐Ahmed et al., 2017 [28]	UK	Reverse phase HPLC and mass spectrometry	Pyridoxal 50-phosphate tablet	Room temperature for 24 hours	Stable at room temperature (protected from light) after 24 h, but unstable after 4 h when exposed to light.	—	—
Pramann et al., 2016 [29]	USA	Ultra-performance liquid chromatography time-of-flight mass spectrometry	Aripiprazole tablet	4°C for 91 days	Stable (>90%)	No apparent physical changes	—
Provenza et al., 2014 [30]	Spain	Optical spectroscopy	Sildenafil tablet	4 and 25°C for 90 days	Stable (>90%)	Stable at 25°C but problem of redispersibility at 4°C	Stable
Shoosanglertwijit et al., 2011 [31]	Thailand	HPLC	Furosemide tablet	Glass bottles are protected from light and stored for 60 days at 4 ± 2°C room temperature (30 ± 2°C) and 3 controlled temperatures of 45°C	Stable (93%)	There was no significant alteration in the appearance (color and consistency) or odour of either formulation	Stable
Soliman et al., 2014 [32]	Egypt	HPLC	Gabapentin tablet	Absence of light at 2–8°C in the refrigerator for 90 days	Stable	—	—
Sosnowska et al., 2009 [33]	Poland	HPLC	Enalapril tablet	Absence of light at 4 and 25°C for 30 days	Stable (98%)	No appreciable changes from the initial pH and initial viscosities	—
Van Der Vossen et al., 2016 [34]	Netherlands	HPLC-UV	Amlodipine tablet	4°C, 25°C, and 40°C for 24 hours	At 25°C and 40°C, subsequent chemical degradation, but it is stable at 4 ^0^C	Stable	—
Zahálka et al., 2018 [35]	Czech Republic	HPLC	Furosemide	25°C ± 3°C or at 40°C ± 0.5°C for 9 months	Stable (>90%)	No color, taste, and odour change	Stable
Zaid, 2016 [36]	Palestine	UV-spectrophotometry	Paracetamol tablet	25°C and 40°C for 4 months	Stable (>93%)	No change in organoleptic properties was observed	Stable

NB: dash (—) in the table indicates that data is not available.

## Data Availability

The data used to support the findings of this study are included within the article.
